# Complexity-Based Decoding of the Coupling Among Heart Rate Variability (HRV) and Walking Path

**DOI:** 10.3389/fphys.2020.602027

**Published:** 2020-11-25

**Authors:** Shahul Mujib Kamal, Mohammad Hossein Babini, Ondrej Krejcar, Hamidreza Namazi

**Affiliations:** ^1^School of Engineering, Monash University Malaysia, Selangor, Malaysia; ^2^Center for Basic and Applied Research, Faculty of Informatics and Management, University of Hradec Králové, Hradec Králové, Czechia

**Keywords:** walking path, sample entropy, heart rate variability, complexity, fractal exponent

## Abstract

Walking is an everyday activity in our daily life. Because walking affects heart rate variability, in this research, for the first time, we analyzed the coupling among the alterations of the complexity of walking paths and heart rate. We benefited from the fractal theory and sample entropy to evaluate the influence of the complexity of paths on the complexity of heart rate variability (HRV) during walking. We calculated the fractal exponent and sample entropy of the R-R time series for nine participants who walked on four paths with various complexities. The findings showed a strong coupling among the alterations of fractal dimension (an indicator of complexity) of HRV and the walking paths. Besides, the result of the analysis of sample entropy also verified the obtained results from the fractal analysis. In further studies, we can analyze the coupling among the alterations of the complexities of other physiological signals and walking paths.

## Introduction

The analysis of heart reactions in different conditions is a very important topic in physiology. Walking is an important action of humans which affects their heart rate variability (HRV).

For this purpose, many researchers analyzed HRV while human walks. They employed different techniques for their analysis. The reported studies that analyzed the effects of regular walking during a golf game (Parkkari et al., 2000), graded forward and backward walking (Hooper et al., 2004), dog-walking (Motooka et al., 2006), low-intensity exercise (Brenner et al., 2020), age and sex of subjects (Corrêa et al., 2013), speed and duration of walking (Saevereid et al., 2014), green walking (de Brito et al., 2020), and supervised walking (Leicht et al., 2011) on heart rate variations are worthy of being mentioned.

In this work, for the first time, we evaluated the coupling between the alterations of heart activity and walking paths. The novelty of our work is employing the concept of complexity to analyze the coupling between walking path and heart reaction. Considering a system that contains many parts that interact with each other in highly different ways, the concept of complexity is used to characterize the behavior of this system. Based on the literature, various techniques have been developed and utilized for the analysis of complex systems.

A human can walk on a straight line or a path with a complex pattern. Moreover, the HRV (in the form of the R-R time series) has a complex pattern. Therefore, complexity theory can be used to study the coupling among HRV and paths. In this study, we used the fractal theory to quantify the complexity of HRV and paths.

The fractal theory is a popular technique for the investigation of complex structures of fractals. In general, fractal objects have repeating patterns (self-affinity or self-similarity) that are distributed on every scale inside them. The complexity of these objects is quantified by the fractal dimension. A more complex object has a larger fractal dimension. In general, for a fractal object, the fractal dimension (as the measure of complexity) satisfies the Szpilrajn inequality:

(1) F≥D

where *F* and *D* represent the fractal dimension and topological dimension (Euclidean dimension) of the object, respectively.

Many works have been reported that investigated the alterations in the complexity of physiological time series using fractal analysis. We can also find some works which applied fractal analysis on ECG signals in various conditions. The reported investigations that analyzed heart rate of normal subjects in different age groups (Acharya et al., 2004; Soares-Miranda et al., 2014), predicted cardiac death (Mäkikallio et al., 2001; Sen and McGill, 2018), evaluated the effects of pharmacological adrenergic and vagal modulation on heart rate dynamic (Tulppo et al., 2001), analyzed the variations of heart rate during non-REM sleep (Togo and Yamamoto, 2001), investigated the variations of heart rate for patients with peripheral arterial disease (Utriainen et al., 2018), diabetes (Chau et al., 1993), and chronic obstructive pulmonary disease (COPD) (D’Addio et al., 2007) using fractal theory are worthy of being mentioned.

Besides fractal analysis, other non-linear analysis techniques such as approximate entropy and sample entropy also can be used to evaluate the complex structure of heart rate. Sample entropy as a measure of complexity does not depend on the data length. Because the heart rates of different subjects have different lengths during walking on the same path, the calculation of sample entropy helps us to verify the result of the fractal analysis which is dependent on the length of data. Sample entropy has been utilized widely to quantify the complexity of various types of physiological signals. Specifically, the applications of sample entropy in the analysis of heart rate have been extensive. The reported works that analyzed complexity change in cardiovascular disease (Chen et al., 2017), investigated the HRV of neonates (Lake et al., 2002), predicted an ischemic stroke in patients with permanent atrial fibrillation by analysis of heart rate (Watanabe et al., 2015), analyzed heart rate variability of children after cardiac transplantation (Tuzcu and Nas, 2005), investigated the recovery of heart rate after exercise (Javorka et al., 2002), classified the heart rate variability of healthy subjects versus subjects with obstructive sleep apnea syndrome (Al-Angari and Sahakian, 2007), and investigated the effect of low-intensity exercise (Weippert et al., 2014) and severity of gastric cancer on HRV (Shi et al., 2019) are worthy of being mentioned.

Therefore, we employed the fractal theory and sample entropy to evaluate the coupling among the complexities of heart rate and walking paths. In the next section, we talk about the methodology. Then, the procedures of data collection and processing will be presented. The result section will provide the findings. Finally, we will discuss the results.

## Materials and Methods

In this work, we evaluated the coupling among the complexities of the HRV and paths of movement. In other words, we analyzed how the alteration in the complexity of a path affects the complexity of the HRV. Therefore, we employed fractal theory and analyzed the alterations of the fractal exponent of the HRV versus the alterations of the fractal exponent of the path of movement. Its larger values indicate greater complexity.

In this study, we considered the R-R time series (obtained by extracting R peaks of ECG signals), as the heart rate. We calculated the fractal exponent of the R-R time series and walking path using the box-counting method. As Eq. 2 shows, the fractal dimension is computed based on the variations of the number (*N*) and size (ε) of boxes used in each iteration of the box-counting algorithm.

(2) $$F\,D=\lim_{\varepsilon \to 0}\frac{\log_{}N\,\left(\varepsilon \right)}{\log1/\varepsilon }$$

Equation 3 shows the general form of fractal dimension (order of *c*) (Soundirarajan et al., 2020):

(3) $$F\,D_{c}=\lim_{\varepsilon \to 0}\frac{1}{c-1}\,\frac{\log_{}\,\sum_{j=1}^{N}r_{j}^{c}}{\log\varepsilon }$$

*r*_*j*_which stands for the probability, is defined as

(4) $$r_{j}=\lim_{T\to \infty }\frac{t_{j}}{T}$$

where *t*_*j*_*and* represent the total time of occurrence in the *j*-th bin and the total period of the time series, respectively.

On the other hand, considering the same walking path, the extracted R-R time series for different subjects had different lengths. To overcome the effect of this issue, and therefore verify the result of fractal analysis, we computed the sample entropy of HRV. Similar to the fractal exponent, a bigger value of sample entropy indicates higher complexity. Therefore, sample entropy was employed to verify the findings of the fractal analysis.

Considering a signal in the form of {*r*(1),*r*(2),*r*(3),…,*r*(*n*)}with a constant interval of α, we define a template vector of length *z*embedding dimension) in the form of *R*_*z*_(*i*) = {*r*_*i*_,*r*_*i* + 1_,*r*_*i* + 2_,…,*r*_*i* + *z*−1_}and the distance function *d*[*R*_*z*_(*i*),*R*_*z*_(*j*)](*i*≠*j*)s to be Chebyshev distance. Then, the sample entropy (*SamEn*) is formulated as

(5) $$S\,a\,m\,E\,n=-\text{log}\,\frac{B}{C}$$

Considering ε as the tolerance (0.2×*standarddeviationofdata*), *B* stands for the number of template vector pairs that

(6) $$d\,\left[R_{z+1}\,\left(i\right),R_{z+1}\,\left(j\right)\right]<\varepsilon$$

Besides, *C* stands for the number of template vector pairs that

(7) $$d\,\left[R_{z}\,\left(i\right),R_{z}\,\left(j\right)\right]<\varepsilon$$

**FIGURE 1:**
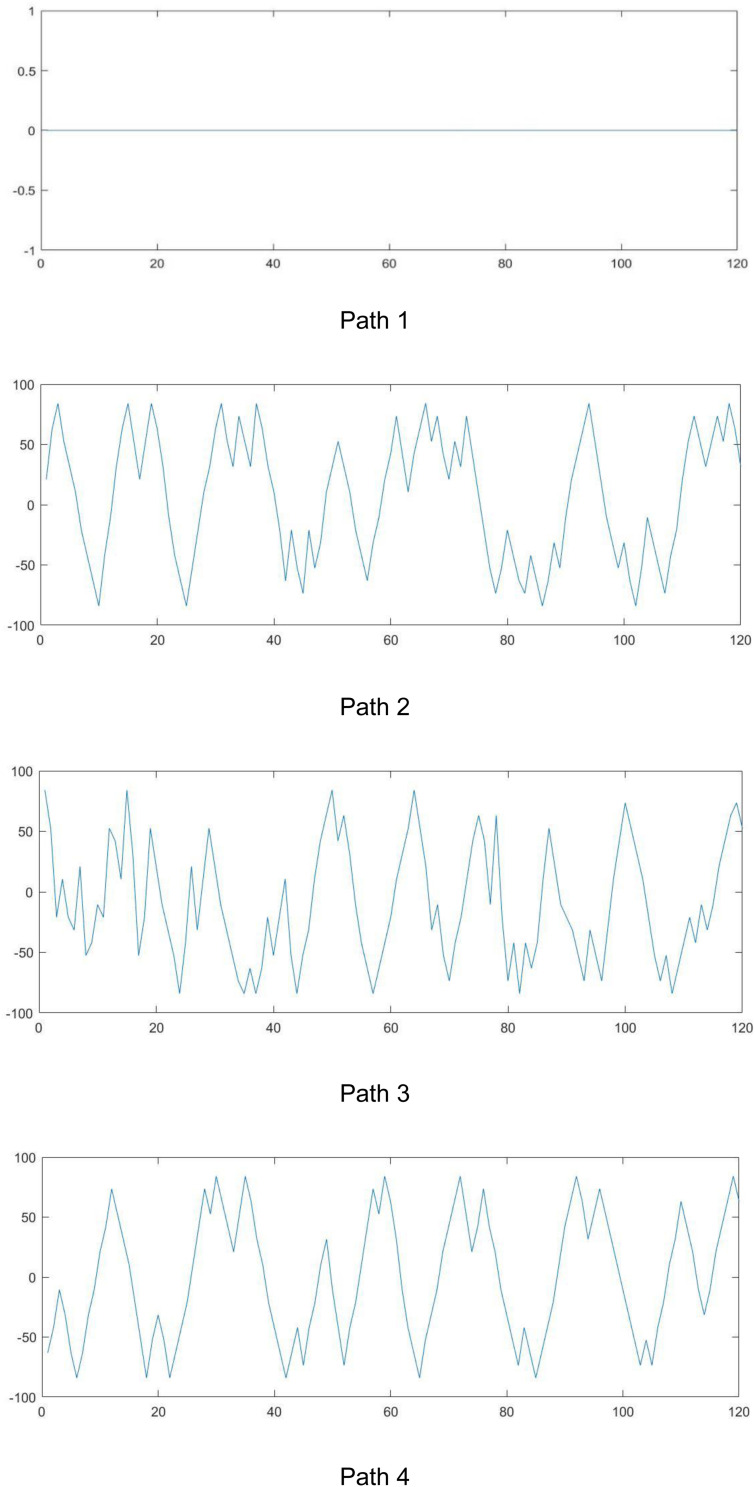
The designed paths. The direction of walking was from left to right in each path.

We designed four walking paths for our experiment that are shown in Figure 1. Each path included 120 points that subjects put their feet on them. The direction of walking was from left to right in each path. We designed these paths based on their fractal exponents. As Figure 1 and Table 1 show, the first path as a straight line has a complexity of 1. By moving to other paths, the complexity of paths increases.

**TABLE 1:** The complexity of various paths.

Number	Fractal exponent
1	1.0000
2	1.6381
3	1.8826
4	1.9771

Therefore, subjects walked on various paths and accordingly, we analyzed the coupling among the alterations of the complexity of the HRV and paths.

### Data Collection and Analysis

The ethical committee of Monash University has approved this study (No. 19719). We have experimented on nine healthy students (6 M, 3 F, 18–22 years old). They signed an informed consent form after they agreed to participate.

For the recording of ECG signals, we used the Shimmer ECG device. Because the shimmer ECG kit is a mobile device, it gave us the ability to record subjects’ ECG signals while walking. We recorded ECG signals from participants with a sampling frequency of 256 Hz. Four recording electrodes and one reference electrode of the ECG device were put on each subject’s chest based on the map that is shown in figure 3-3 in Figure 3-3 (2019).

The experiment has been conducted in two similar sessions. In each session, we asked participants to look through and walk on the designed points on the paths without doing extra movements or looking around. Initially, we recorded the ECG signals of subjects while resting for 1 min. After that, participants walked on the first to the fourth path, while we recorded their ECG signals. It should be noted that they rest for a minute (by sitting on a chair) once they reached the last point of each path. This period brought their heart activity to the normal condition before they move on another path.

**FIGURE 2:**
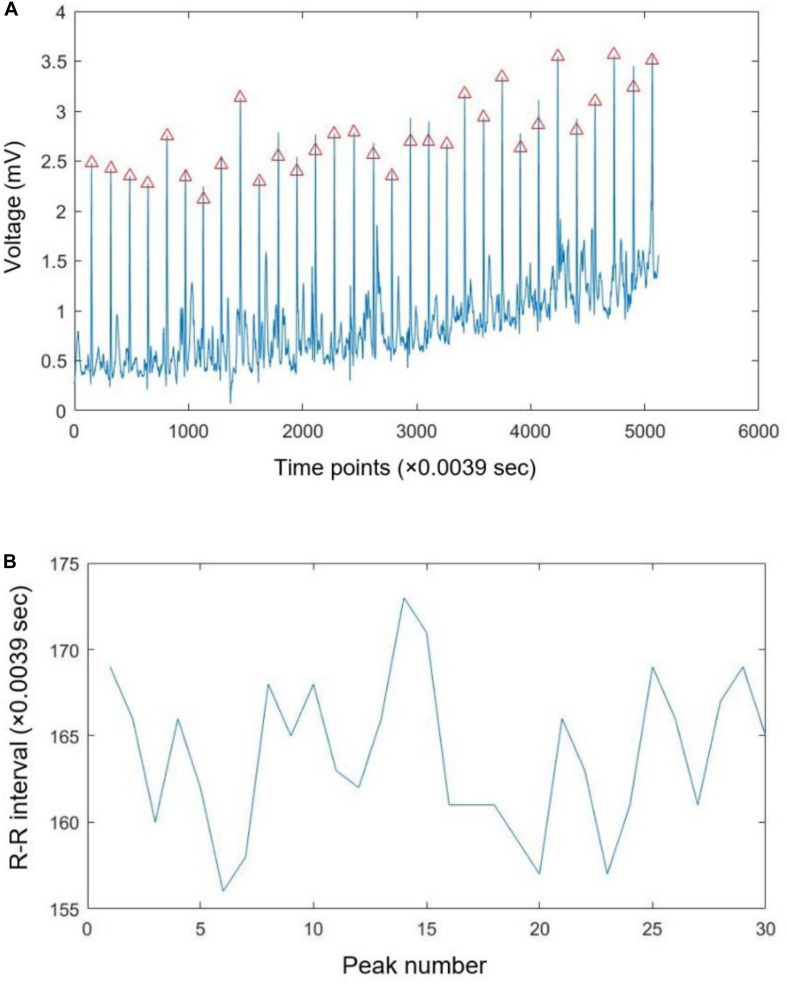
A sample of recorded ECG signal with the specified R peaks **(A)** and extracted R-R time series **(B)**.

For our analysis, we considered the R-R time series as the heart rate variability (HRV) signal. For this purpose, we wrote a set of codes in MATLAB that generated the R-R time series. These written codes detected R peaks through QRS analysis and accordingly generated R-R time series. For this purpose, our code first de-trended ECG signals using fast Fourier transform and accordingly transformed the result to time-domain via inverse fast Fourier transform. After that, our code used “*findpeaks*” command in MATLAB to find the peaks based on the minimum specified voltage, and the minimum distance of each two consecutive peaks. Figure 2 shows a sample of raw ECG signal (A) and extracted the R-R time series for 20 s of data (5,120 sample points) (B). It should be noted that we visually checked all selected peaks to ensure their correctness.

We processed the R-R time series in case of different walking paths by computing their fractal exponent and sample entropy in MATLAB 2019a (MathWorks, United States). The duration of walking for each subject was different in the case of various paths; therefore, the length of his/her R-R time series was different. However, we processed the same length of data (58.19 s) for all subjects in case of rest. A code based on the box-counting algorithm calculated the fractal dimension of R-R time series using boxes with the sizes of $\frac{1}{2},\frac{1}{4},\frac{1}{8},\frac{1}{16},\ldots .$ the minimum box size is calculated in the box-counting algorithm (Han, 2020).

Initially, we checked the assumption of the ANOVA test (normality, equality of variances, and independence). After that, the ANOVA test was conducted to evaluate the significance of alterations of the complexity of HRV due to walking. We compared the fractal exponent (and sample entropy) of HRV among various conditions (rest and walking paths) using a *post hoc* Tukey test. The effect of variations of paths on the alterations of the complexity of HRV was investigated u sing effect size analysis, and Cohen’s *d* has been reported. We also analyzed the coupling between the calculated values of fractal exponent and sample entropy using the Pearson correlation coefficient. We interpreted the results based on the significance level of 95%.

## Results

**FIGURE 3:**
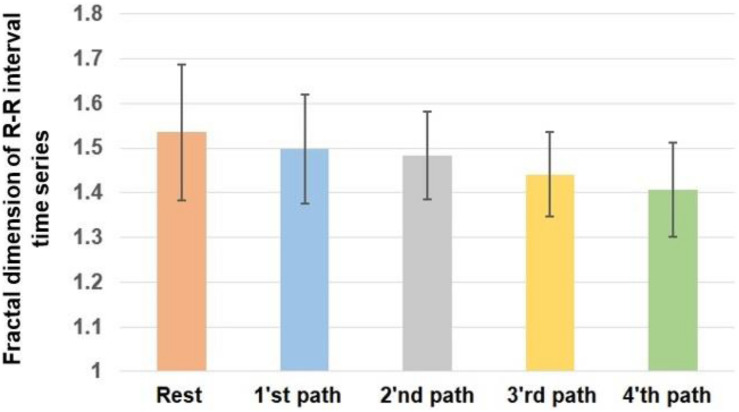
The fractal exponent of heart rate variability. Error bars indicate the SD.

**FIGURE 4:**
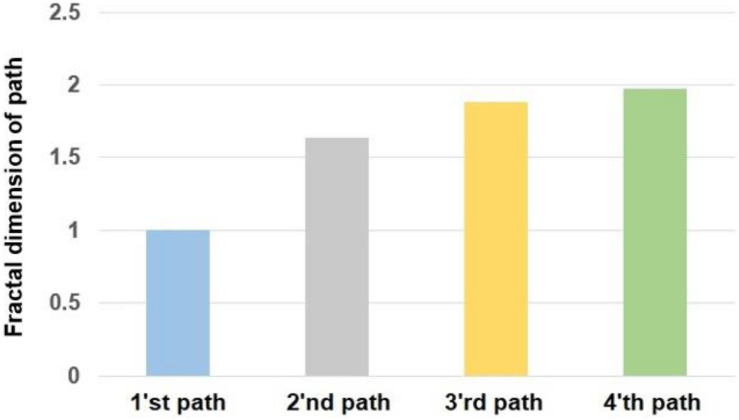
The fractal dimension of different paths.

The assumptions of the ANOVA test for the calculated values of fractal exponent and sample entropy have been fulfilled. The presented results are based on the average of calculated values in two sessions of the experiment. The alterations of the fractal exponent of the R-R time series are shown in Figure 3. For a better comparison, we mapped the fractal exponent of paths in Figure 4.

*P* value = 0.0320 and *F* value = 2.7975 calculated from the ANOVA test indicate that the fractal dimension of heart rate has changed significantly as the result of walking. According to the obtained results, the heart rate has the largest fractal exponent during rest. The fractal exponent indicates the complexity; therefore, the heart rate experiences the greatest complexity during rest. The trend of alterations of the fractal exponent shows that shifting from first to the fourth path reduced the complexity of heart rate.

By comparing the obtained results in Figure 3 with the complexity of various paths in Figure 4, it can be said that the complexity of heart rate decreased when participants moved on a path with a larger complexity. Therefore, the alterations of the complexities of HRV and paths are coupled. The value of correlation coefficient (*R* = −0.8657) between the variations of the fractal exponents of HRV and walking path also proves a strong negative coupling between them.

Table 2 compares the fractal exponent of heart rate among various pairs. As is shown, the alteration of the fractal exponent of heart rate among rest and the fourth path was significant. In fact, the difference in the complexity of paths affects the result of pairwise comparisons, and greater differences between the complexities of paths would potentially cause significant differences in the fractal exponents of HRV. This table also shows the effect sizes. As it is clear, the fourth path with the largest complexity had the largest influence on the alterations of the complexity of heart rate.

**TABLE 2:** Comparing the fractal exponent of HRV.

Pair	*p*	Cohen’s *d*
Rest condition and 1st path	0.8989	0.2660
Rest condition and 2nd path	0.6937	0.4090
Rest condition and 3rd path	0.1343	0.7446
Rest condition and 4th path	0.0293	0.9801
1st path and 2nd path	0.9958	0.1422
1st path and 3rd path	0.6391	0.5292
1st path and 4th path	0.2491	0.8049
2nd path and 3rd path	0.8433	0.4351
2st path and 4th path	0.4218	0.7453
3rd path and 4th path	0.9340	0.3396

**FIGURE 5:**
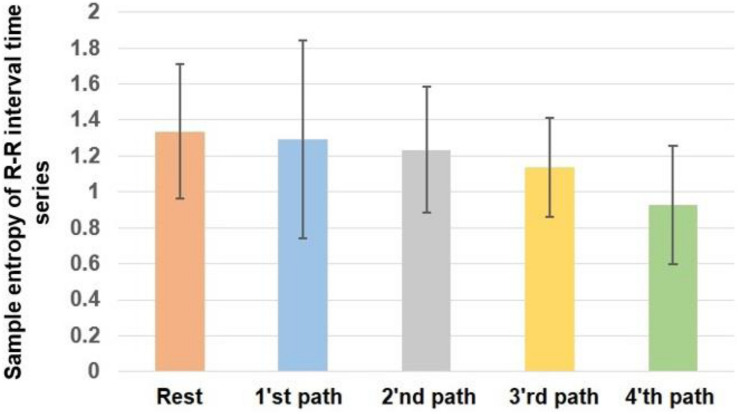
The sample entropy of heart rate variability. Error bars indicate the SD.

Figure 5 demonstrates the alterations of the sample entropy of the R-R time series in rest and various paths.

*P* value = 0.0455 and *F* value = 2.5599 calculated from the ANOVA test indicate that the sample entropy of heart rate has changed significantly as the result of walking. Based on the obtained results, the heart rate has the highest sample entropy in case of rest. Therefore, it can be said that the heart rate experiences the greatest complexity during rest. The trend of alterations of the sample entropy in the case of different paths indicates that by shifting from first to the fourth path, the complexity of heart rate decreases.

By comparing the obtained results in Figure 5 with the complexity of different walking paths shown in Figure 4, it can be said that by moving on a path with greater complexity, the complexity of heart rate decreased. Therefore, the trend of alterations of sample entropy is similar to the trend of alterations of the fractal exponent.

Table 3 compares the sample entropy of heart rate between various conditions. Based on this result, similar to what we observed in Table 2, the alterations of the sample entropy of heart rate among rest and the fourth path was significant. As previously mentioned, the difference in the complexity of paths affects the result of pairwise comparisons, and greater differences between the complexities of paths would potentially cause significant differences in the sample entropy of HRV.

**TABLE 3:** Comparing the sample entropy of HRV.

Pair	*p*	Cohen’s *d*
Rest condition and 1st path	0.9978	0.0907
Rest condition and 2nd path	0.9398	0.2786
Rest condition and 3rd path	0.5526	0.6011
Rest condition and 4th path	0.0363	1.1622
1st path and 2nd path	0.9932	0.1269
1st path and 3rd path	0.7854	0.3572
1st path and 4th path	0.0989	0.8104
2nd path and 3rd path	0.9507	0.3067
2st path and 4th path	0.2135	0.9079
3rd path and 4th path	0.5731	0.6972

The effect sizes are presented in this table. As it is clear, the fourth path with the largest complexity had the largest influence on the alterations of the complexity of heart rate. Moreover, the value of correlation coefficient (*R* = 0.9588) between the variations of sample entropy and fractal exponent indicates a strong positive correlation between them.

Therefore, the result of the analysis of sample entropy of heart rate validates the findings of fractal analysis. In general, the alterations of the complexities of HRV and walking path are coupled; as subjects walked on a path with a larger complexity, a larger change was seen in the complexity of their heart rates.

## Discussion and Conclusion

We evaluated the effect of walking on different paths on HRV. For this purpose, for the first time, we considered the concept of complexity, and by employing fractal theory and sample entropy, we studied the alterations of the complexity of heart rate in case of walking on various paths with various complexities. The findings showed greater alterations in the complexity of heart rate as the result of walking on paths with greater complexities. In other words, the complexities of the heart rate and walking paths are coupled. The result of statistical analysis demonstrated significant alterations in the complexity of HRV due to walking on the various paths. Moreover, moving on a path with a larger complexity had a larger effect on the alterations of the HRV.

Our analysis is more advanced compared with the studies (Parkkari et al., 2000; Hooper et al., 2004; Motooka et al., 2006; Leicht et al., 2011; Corrêa et al., 2013; Saevereid et al., 2014; Brenner et al., 2020; de Brito et al., 2020) that only evaluated HRV in walking without relating it to the characteristics of the walking path. Besides, decreasing the complexity of HRV in walking compared with the rest has been observed in Shi et al. (2017), and therefore, the result of our analysis is valid.

Here, we refer to the connection between the brain and heart to elaborate on the obtained results in this study. In Kamal et al. (2020), we showed that the alterations of the complexities of EEG signals and walking paths are coupled. Because the human brain regulates the heart activity through the physiological network, therefore, the alterations of the complexity of EEG signals are mapped on the alterations of the complexity of HRV. In other words, the complexity of EEG (Kamal et al., 2020) and HRV experiences larger alterations when participants move on paths with larger complexities.

In this work, we evaluated the variations of HRV during walking. In further studies, similar experiments can be performed in case of other physiological signals. For example, we can analyze how respiration signals change while walking on different walking paths. We can simultaneously analyze the brain’s reaction while subjects walk on different paths. As was mentioned previously, because the human brain regulates all activities of the body, couplings should exist among the alterations of the complexities of EEG signals and other biomedical signals in walking. For example, we can evaluate the coupling among the alterations of EEG, ECG, and moving paths. This analysis will help us to evaluate the coupling among brain and heart activities during walking that specifically has great importance in rehabilitation science.

Besides, our analysis can be further extended for patients with various heart disorders. Therefore, we can evaluate the coupling among the complexities of HRV and moving paths in the case of these patients. Accordingly, we can understand how a disorder affects heart activity, and by adjusting paths, we can regulate heart reactions. In other words, we can design the path in which patients can walk with fewer problems for their hearts.

## Data Availability Statement

The raw data supporting the conclusions of this article will be made available by the authors, without undue reservation.

## Ethics Statement

The studies involving human participants were reviewed and approved by the Monash University Human Research Ethics Committee (MUHREC). The patients/participants provided their written informed consent to participate in this study.

## Author Contributions

HN designed the study, supervised the experiment, did the data analysis, and wrote the manuscript. MB ran the experiment and did the data analysis. SM ran the experiment. OK verified the result and helped in revising the manuscript. All authors contributed to the article and approved the submitted version.

## Conflict of Interest

The authors declare that the research was conducted in the absence of any commercial or financial relationships that could be construed as a potential conflict of interest.
